# Functional and Molecular Heterogeneity in Glioma Stem Cells Derived from Multiregional Sampling

**DOI:** 10.3390/cancers15245826

**Published:** 2023-12-13

**Authors:** Marit Brynjulvsen, Elise Solli, Maria Walewska, Manuela Zucknick, Luna Djirackor, Iver A. Langmoen, Awais Ahmad Mughal, Erlend Skaga, Einar O. Vik-Mo, Cecilie J. Sandberg

**Affiliations:** 1 Vilhelm Magnus Lab, Institute for Surgical Research and Department of Neurosurgery, Oslo University Hospital, Nydalen, P.O. Box 4950, 0424 Oslo, Norway; 2 Institute of Clinical Medicine, Faculty of Medicine, University of Oslo, Blindern, P.O. Box 1112, 0317 Oslo, Norway; 3 Department of Biostatistics, Oslo Centre for Biostatistics and Epidemiology, University of Oslo, Blindern, P.O. Box 1122, 0317 Oslo, Norway

**Keywords:** glioblastoma, intratumoral heterogeneity, glioblastoma stem cells, drug sensitivity and resistance testing

## Abstract

**Simple Summary:**

Glioblastoma, the most common and malignant brain tumor, is driven by a population of stem and progenitor cells. We have recently demonstrated that identification of novel drug treatment options by screening glioblastoma stem cell cultures is feasible within a clinical timeframe. We have also identified significant interheterogeneity between glioblastoma stem cell cultures both on the molecular and functional level. Here, we present that glioblastoma stem cell cultures derived from spatially distinct regions representing one entire tumor mass share typical stem cell properties and global gene expression profiles but display considerable differences in somatic mutations and drug sensitivity profiles. However, the heterogeneity within regional glioblastoma stem cell cultures is less complex than what is found between tumors from different patients. Our results suggest that single, focal biopsies used to establish patient-derived GSC cultures may underestimate the total complexity of the glioblastoma stem cell population.

**Abstract:**

Glioblastoma (GBM) is an aggressive and highly heterogeneous primary brain tumor. Glioma stem cells represent a subpopulation of tumor cells with stem cell traits that are presumed to be the cause of tumor relapse. There exists complex tumor heterogeneity in drug sensitivity patterns between glioma stem cell (GSC) cultures derived from different patients. Here, we describe that heterogeneity also exists between GSC cultures derived from multiple biopsies within a single tumor. From biopsies harvested within spatially distinct regions representing the entire tumor mass, we established seven GSC cultures and compared their stem cell properties, mutations, gene expression profiles, and drug sensitivity patterns against 115 different anticancer drugs. The results were compared to 14 GSC cultures derived from other patients. Between the multiregional-derived GSC cultures, we observed only minor differences in their phenotype, proliferative capacity, and global gene expression. Further, they displayed intratumoral heterogeneity in mutational profiles and sensitivity patterns to anticancer drugs. This heterogeneity, however, did not exceed the extensive heterogeneity found between GSC cultures derived from other GBM patients. Our results suggest that the use of GSC cultures from one single focal biopsy may underestimate the overall complexity of the GSC population and display the importance of including GSC cultures reflecting the entire tumor mass in drug screening strategies.

## 1. Introduction

Glioblastoma (GBM) is a devastating form of primary brain cancer. In unselected patients, the median survival is less than a year [1] and rises to around 15 months in patients treated with surgery and radio- and chemotherapy [2]. An imperative factor that contributes to treatment resistance is intertumoral heterogeneity between individual patients at the molecular, cellular, and functional level [3,4]. The considerable molecular differences existing within single tumors—the intratumoral heterogeneity (ITH)—adds additional complexity [5]. In GBM, ITH in genetic aberrations, transcriptomic profiles, and DNA methylation patterns is described both at the regional and single-cell level [6,7,8,9,10,11,12]. Thus, the subgrouping of patients based on genetic or transcriptional profiling has proven to be of limited value, as different areas within the same tumor may classify into different subgroups [10,13].

At the cellular level, a subpopulation of GBM cells—glioma stem cells (GSCs)—share phenotypic traits with normal neural stem cells [14,15]. These cells represent the top of an evolutional hierarchy in GBM that upon serial xenotransplantation can reconstruct the entire cellular spectrum of a GBM along with highly infiltrative growth [3]. We have previously shown that patient-derived glioma stem cell (GSC) cultures maintain their ability to form invasive tumors, preserve patient-specific traits, and display functional characteristics that have clinical predictive value for patients, thus representing an individualized model of its parent tumor [14,16,17].

Although a complex tumor heterogeneity in GBM is well described, there is still limited understanding of the heterogeneity in the GSC subpopulation. A few recent studies have explored the heterogeneity of GSCs at the single cell [18,19], microenvironmental [20,21], and epigenetic level [22]. Three studies have explored ITH in GSC cultures derived from single cell clones and described variations in morphology, growth pattern, differentiation capacity, and drug sensitivity [11,12,23]. However, these studies explore GCS from randomly harvested single cells, leaving uncertainty as to whether their findings reflect GSC heterogeneity across the entire tumor mass.

To develop tailored treatments to individual GBM patients, there is increasing interest in using patient-derived cells and drug screening technologies for functional precision medicine [24]. We have previously described the intertumoral heterogeneity of patient-derived GSC cultures from both treatment-naïve and recurrent GBMs in terms of drug sensitivity patterns to anticancer drugs [4,25]. These GSC cultures were established from a collection of tumor biopsies and the ultrasonic aspirate generated during surgery. In this study, our aim was to explore whether ITH exists in GCS cultures derived from multiregional biopsies from the same tumor and to compare drug responses’ ITH to the heterogeneity found between different tumors. 

## 2. Materials and Methods

### 2.1. Brain Tumor Biopsies and Cell Cultures

Biopsies were obtained from informed and consenting patients undergoing surgery for GBM at Oslo University Hospital, Norway, according to the Helsinki declaration. The project was approved by the Norwegian Regional Committee for Medical and Health Research Ethics (REK 2016/1791 and 2017/167). Histopathological diagnostics were performed according to the 2016 WHO classification and all patients were IDHwt. For the multiregional samples (T20-088), six focal and multiregional tumor biopsies were performed (biopsy I to VI), and tumor tissue was harvested through cavitron ultrasonic aspiration (Söring GmbH, Quickborn, Germany) during surgery (ultrasonic aspirate, UA). 

The focal and multiregional biopsies were predominantly derived from specifically localized tumor edges, whilst the UA included cells from multiple parts of the tumor core and periphery. The tumor biopsies were dissociated and cultured in serum-free, growth factor-enriched media for the enrichment of GSCs, as previously described [17]. The GSC cultures were validated for stem cell properties including sphere-forming abilities, differentiation capacity (immunofluorescent staining using mouse-β-3-tubulin and rabbit-GFAP, Merck Life Science AS, Darmstadt, Germany) and the expression of stem cell surface markers (flow cytometry analysis using CD15-PerCP, R&D Systems, Abingdon, UK; CD44-APC, AH diagnostics as, Oslo, Norway; CD133-PE, Miltenyi Biotec, Lund, Sweden; CXCR4-PE, Miltenyi Biotec), as previously described [4]. 

### 2.2. DNA and RNA Sequencing

DNA and RNA were studied by next-generation sequencing. Simultaneous DNA and RNA isolation from a single cell pellet was conducted using AllPrep DNA/RNA Micro Kit (Qiagen, Qiagen GmbH, Hilden, Germany) following the manufacturer’s protocol. Cells were collected for DNA and RNA isolation at passage two following dissociation. DNA was also harvested from patient blood leucocytes and isolated following the AllPrep DNA/RNA Micro Kit (Qiagen) procedure and sequenced to exclude germline mutations. DNA from both GSC cultures and blood leucocytes was sequenced using the Illumina platform HiSeq4000 (Illumina^®^, San Diego, CA, USA). RNA was sequenced using the Illumina platform Next Seq500 (Illumina^®^, San Diego, CA, USA) and conducted by the Genomics and Bioinformatics Core Facility at the Norwegian Radium Hospital, Oslo University Hospital.

The exome sequencing data were processed using Illumina Dragen and annotations of the called variants were processed by using Cancer Predisposition Sequencing Reporter and Personal Cancer Genome Reporter [26,27]. The processing of RNA sequencing data was conducted using the Tuxedo pipeline (Cufflinks Assembly and DE (BaseSpace Workflow) 2.1.0, Isis (Analysis Software) 2.6.25.18, STAR (Aligner) STAR_2.5.0b, Isaac Variant Caller 2.3.13-31-g3c98c29-dirty, BEDTools 2.17.10, Cufflinks 2.2.1, and BLAST 2.2.26+). Gene expression was expressed as transcripts per million (TPM). Genes marked as failed in one or more of the GSC cultures and genes with TPM equal to zero in all samples were not included in further analyses. Identification of heterogeneously and homogenously expressed genes was conducted by fitting a mixture model to the logarithm of the standard deviation of the gene expressions across the different samples in which lowly expressed genes across all samples (average TPM < 1) were discarded. Gene set enrichment analysis was completed using the online tool DAVID (Database for Annotation, Visualization, and Integrated Discovery, Bioinformatics Resources 6.8, Laboratory of Human Retrovirology and Immunoinformatics (LHRI)) [28]. DAVID uses a modification of the Fischer’s Exact test with Benjamini correction at alpha level 0.05 or statistical hypothesis testing. The predicted effects of the mutations were determined using Sorting Intolerant From Tolerant (https://sift.bii.a-star.edu.sg, accessed on 6 November 2023) [29].

### 2.3. Temozolomide Sensitivity Assays

Cells were plated at a density of 5000 cells/well in a 96-well plate under sphere forming conditions. After 24 h, vehicle 0.5% DMSO and temozolomide (TMZ) were added, and the cells were further incubated for 10 days. TMZ concentrations covered a 5-point dose-escalating pattern (0.4–250 µM). Cell proliferation was assessed using the CellTiter-Glo luminescent assay (Promega, Madison, WI, USA). Absorbance was measured using the POLARstar Omega (BMG LABTECH, Ortenberg, Germany) and analysis was conducted using the Omega Data Analysis software v3.10 R6 (BMG LABTECH, Ortenberg, Germany). The resulting data were normalized to negative controls only added dimethyl sulfoxide (DMSO, Merck Life Science AS).

### 2.4. Drug Sensitivity and Resistance Testing

Drug sensitivity and resistance testing (DSRT) was performed as previously described, allowing for a quantitative comparison of individual cultures by one single response metric [30]. The drug collection consisted of 115 different anticancer compounds of both U.S. Food and Drug Administration (FDA)-approved drugs and selected investigational compounds covering a range of drug classes with various mechanisms of action (e.g., conventional chemotherapies, kinase inhibitors, immunomodulatory drugs, epigenetic modifiers, hormone therapy, and apoptotic modulators (Supplementary Table S1). Each drug was tested in a viability assay including a five-point dose-escalating pattern covering a clinically relevant drug range. The raw data were scaled to both negative (DMSO) and positive (100 µmol/L benzethonium chloride) controls to generate relative viability percent inhibition. Individual drug responses were quantified by estimating the Drug Sensitivity Score (DSS), a metric combining the advantages of both the complex dose–response curve (IC50) and the closed-form integration of the area under the curve (AUC) into one single response [30,31]. The calculation was performed using the BREEZE online application [32]. In brief, the five-point dose–response curves were modeled by a four-parameter logistic fit function that was defined by the top and bottom asymptote, the slope, and the IC50. The drug screening was performed when the GSC cultures were at passage eight. To generate a culture-specific DSS, the average DSS for each drug from 14 primary GSC cultures were subtracted from each individual DSS, named selective Drug Sensitivity Scores (sDSS) [4,25,33].

### 2.5. Statistical Considerations

Data analysis and graphic presentation were performed using Microsoft Excel version 2016, GraphPad Prism version 8.0/9.0, R version 4.1.0, and J-Express 2011. Unsupervised hierarchical clustering and heat maps were generated using R 4.1.0 and J-Express 2011. Statistical analysis of global gene expression and drug sensitivity between cultures was performed using visual variance analysis with unsupervised hierarchical clustering, PCA-plots (log2(value + 0.01)), and one-sided statistical F-tests of equality of variances. Correlation analysis was performed using Spearman.

## 3. Results

### 3.1. GSC Cultures Derived from Regionally Distant Biopsies Share Phenotypic Traits

**Figure 1:**
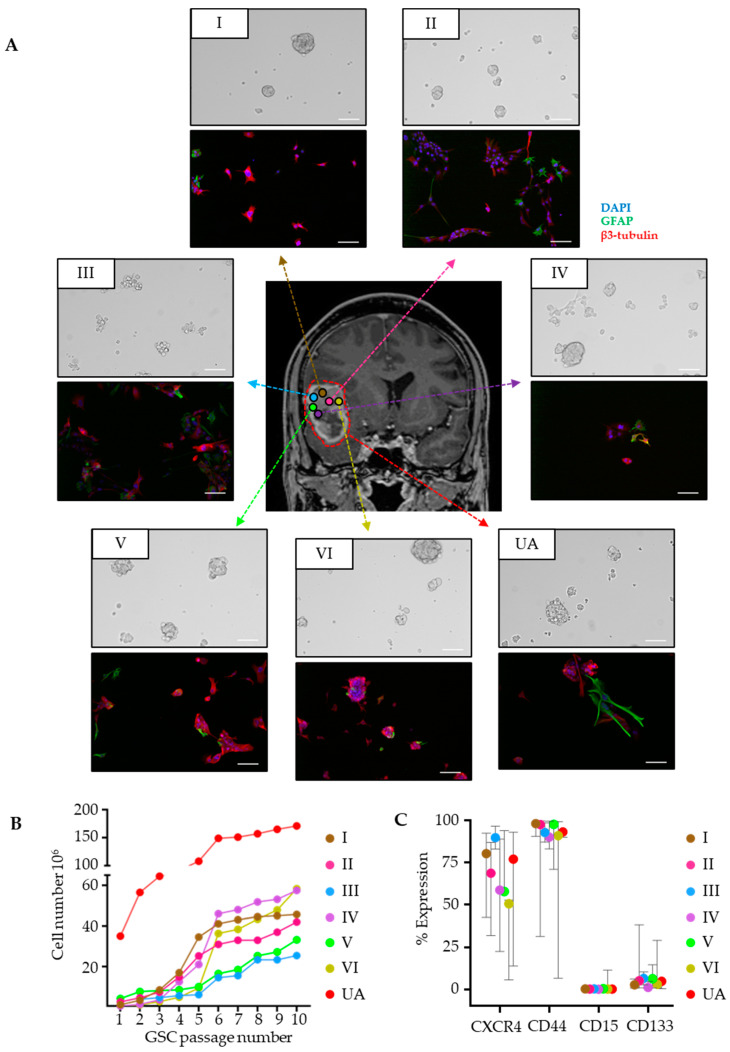
GSC cultures derived from multiregional sampling share phenotype and growth characteristics. (**A**) Schematic illustration of GSC cultures established from focal biopsies (I–VI) and the ultrasonic aspirate harvested during surgery (UA) with respective cell culture and differentiation morphology. Scale bar = 100 µm. Red = β3-tubulin, green = GFAP, and blue = DAPI. (**B**) Growth kinetics of the individual GSC cultures. (**C**) Flow cytometric analysis of stem cell markers in the GSC cultures (n = 3 for each culture) displaying the median with range.

Six focal tumor biopsies (I–VI) and the ultrasonic aspirate (UA) were obtained from one IDH^wt^ GBM. While the focal and multiregional biopsies represented spatially distinct areas of the tumor, the UA was sampled during tumor resection with a cavitron ultrasonic aspirator that collects tissue from multiple parts of the tumor as well as infiltrating the brain (Figure 1A). The cultures proliferated as free-floating spheres, although the culture obtained from location IV proliferated with a semi-adherent morphology (Figure 1A). Following exposure to differentiating conditions, all cultures displayed a more mature morphology and showed positive staining for GFAP and β3-tubulin (Figure 1A). All seven GSC cultures showed a similar growth pattern with accelerated growth onwards from passage four or five (Figure 1B). Further, they expressed high levels of the GSC markers CD44 and CXCR4 but low or no expression of CD15 and CD133 (Figure 1C, Supplementary Table S2). There were no significant differences in expression of any of the GSC-related markers between the cultures (*p* = 0.5, two-way ANOVA). Collectively, we observed only minor differences in the sphere-forming and differentiation abilities, proliferative capacity, and expression of stem cell markers between the GSC cultures. 

### 3.2. ITH in Mutational and Gene Expression Profiles

**Figure 2:**
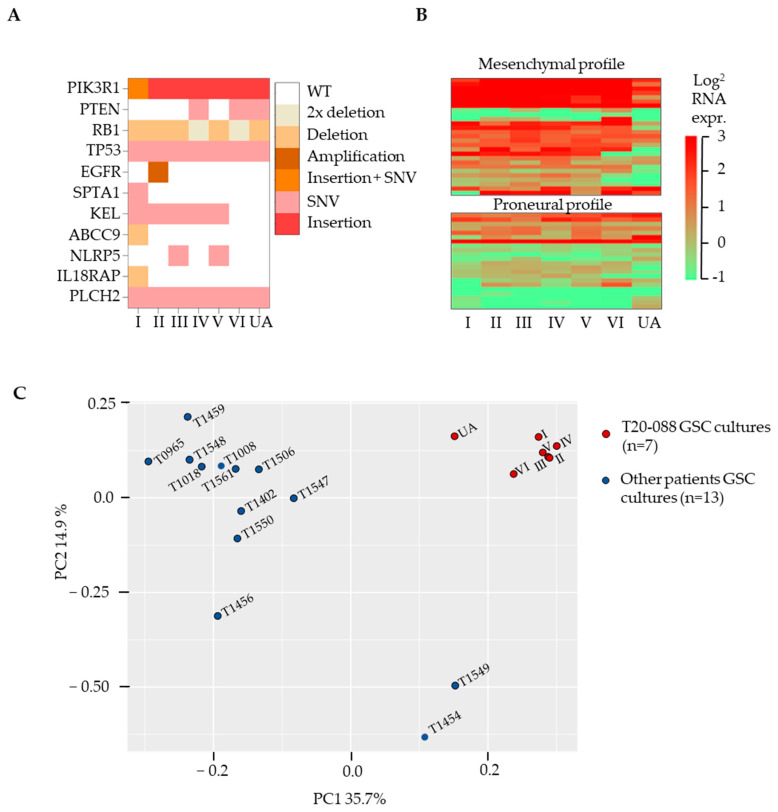
Multiregional tumor sampling displays intratumoral heterogeneity on mutations, while global gene expression is patient specific. (**A**) Selected GBM-associated mutations found in the GSC cultures. (**B**) GSC subtype profiling showing mesenchymal and proneural profile in GSC. Cultures overall express more mesenchymal genes. (**C**) Principal component analysis of GSCs demonstrate clustering of the cultures from the same patient (n = 7) in both the first and second component, indicating less ITH compared to GSC cultures derived from 13 other patients’ IDH_wt_ GBM.

Next, we examined the molecular makeup of the different GSC cultures exploring somatic mutations and global gene expression. To minimize the risk of additional genetic aberrations from extended in vitro culturing, the nucleic acids were extracted from all cultures at passage two. Exome sequencing showed that the GSC cultures had on average 195 cancer-related mutations (range: 180–227) (Supplementary Table S3). We found single-nucleotide variation (SNV) mutation in TP53 and PLCH2 genes present throughout the cultures, the gene mutations described to be involved in GBM pathogenesis (Figure 2A) [34]. The cultures demonstrated heterogeneity in genetic aberrations central to GBM pathogenesis, including PTEN, RB1, and EGFR (Figure 2A). Functional mutation analysis predicted the outcome of the TP53, PTEN, and PLCH2 mutations as damaging, whilst the RB1 alterations were classified as tolerated. 

We found that the cultures were more enriched in gene expression related to the mesenchymal subtype, although UA demonstrated a relative enrichment of proneural gene expression (Figure 2B) [35,36]. To further explore the ITH in gene expression, we fitted a mixed model to the gene expression data and identified genes that were either homogenously or heterogeneously expressed between the seven different cultures. Genes with at least a 99.8% probability of falling into either of the two categories were classified as homogenous or heterogeneous, respectively. This approach identified 4386 genes as homogenously expressed and 424 as heterogeneously expressed (Supplementary Table S4). The most significantly enriched pathways in the homogenous gene sets were related to 68 KEGG pathways including signaling in degenerative diseases and genetic information-processing like the ribosome and the spliceosome (Supplementary Table S5). Only four KEGG pathways were significantly enriched in the heterogeneously expressed gene set. They were related to neuroactive ligand–receptor signaling and cell adhesion, including several MHC II molecules. 

To evaluate the extent of molecular ITH, we compared the global gene expression of the multiregional GSC cultures to 13 individual GSC cultures derived from treatment-naïve IDH^wt^ GBM [4]. The multiregional GSC cultures grouped closely compared to the other cultures in principal component analysis (Figure 2C), suggesting that the ITH of the GSC cultures does not exceed the extensive intertumoral heterogeneity found between patients. This was also supported by correlation analysis (Supplementary Figure S1). 

Collectively, the multiregional GCS cultures shared molecular profiles but with some heterogeneity in gene expression and selected GBM relevant mutations. The ITH was, however, considerably less than the heterogeneity found between individual patient GSC cultures.

### 3.3. ITH in Drug Sensitivity to Anticancer Drugs

We next compared the druggable vulnerabilities in the multiregional GSC cultures by their dose–response score (DSS), a quantitative metric combining the closed-form integration of the area under the curve (AUC) and the dose–response model (IC50) into one single metric [30,33]. First, we evaluated the sensitivity to TMZ, the standard-of-care in GBM treatment. None of the seven multiregional GSC cultures were sensitive to TMZ (average DSS 0.5, range 0–1.8, *p* = 0.38, Supplementary Table S6). This was in concordance with an unmethylated MGMT-promoter of the tumor. We further evaluated the drug sensitivity patterns of the GSC cultures to a panel of 115 anticancer drugs quantified by the DSS (Supplementary Table S7, with corresponding AUC and IC50). 

**Figure 3:**
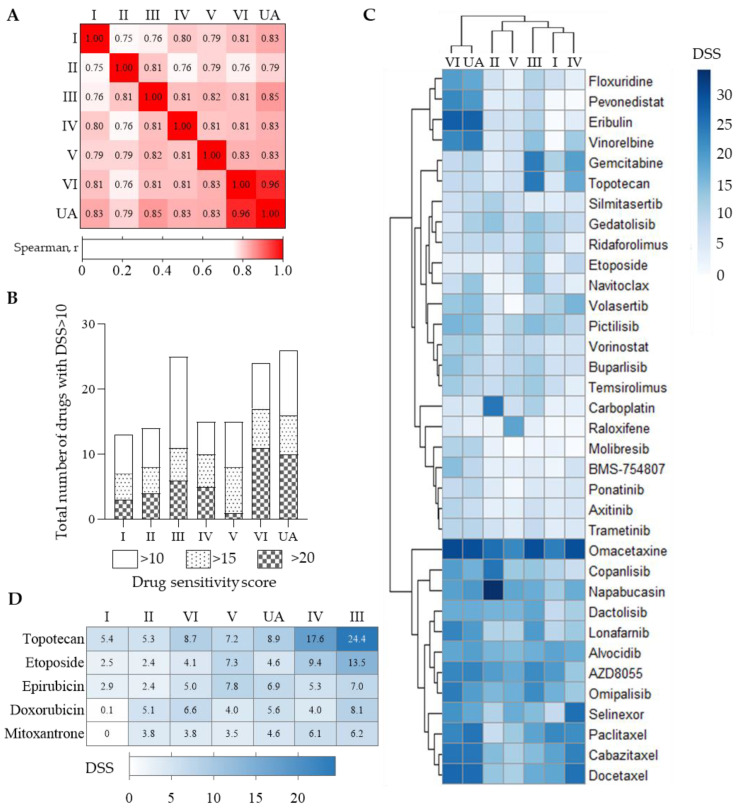
Drug sensitivity screening of GSC cultures (I–VI, UA) derived from multiregional sampling. (**A**) Spearman r correlation analysis of the overall sensitivity to the entire drug collection (n = 115) demonstrates high correlation (>0.75). (**B**) Total number of drugs with DSS ≥ 10 in each culture. (**C**) Unsupervised hierarchical clustering of the multiregional GSC cultures. Drugs selected by DSS ≥ 10 in at least one culture. (**D**) Drug sensitivity patterns to topoisomerase inhibitors in the multiregional GSC cultures demonstrating biological consistency.

The multiregional GSC cultures’ drug sensitivity patterns had overall a strong correlation (Spearman r 0.80±0.08, Figure 3A). VI and UA were highly similar, while culture II deviated from the rest (Figure 3A). We previously defined a DSS ≥ 10 as a threshold to represent a moderate to strong drug response [4]. In the drug collection, 35 drugs displayed a response of DSS ≥ 10 in at least one of the GSC cultures. Three of the GSC cultures (III, VI, and UA) were considerably more sensitive to the drug collection with a higher number of drugs with such a response (Figure 3B). Further, unsupervised hierarchical clustering of these drugs identified VI and UA as a subcluster separately from the rest of the GSC cultures (Figure 3C).

We have earlier described the biological consistency in drug sensitivity patterns within a class of drugs in both the treatment-naïve and recurrent GBM [4,25]. We observed a similar pattern in GSC cultures derived from the same tumor. For instance, the sensitivity to topoisomerase inhibitors was variable between the individual cultures, ranging from sensitive (III) to resistant (I) cultures, but the responses to the class of topoisomerase inhibitors were overall consistent within the individual cultures (Figure 3D).

**Figure 4:**
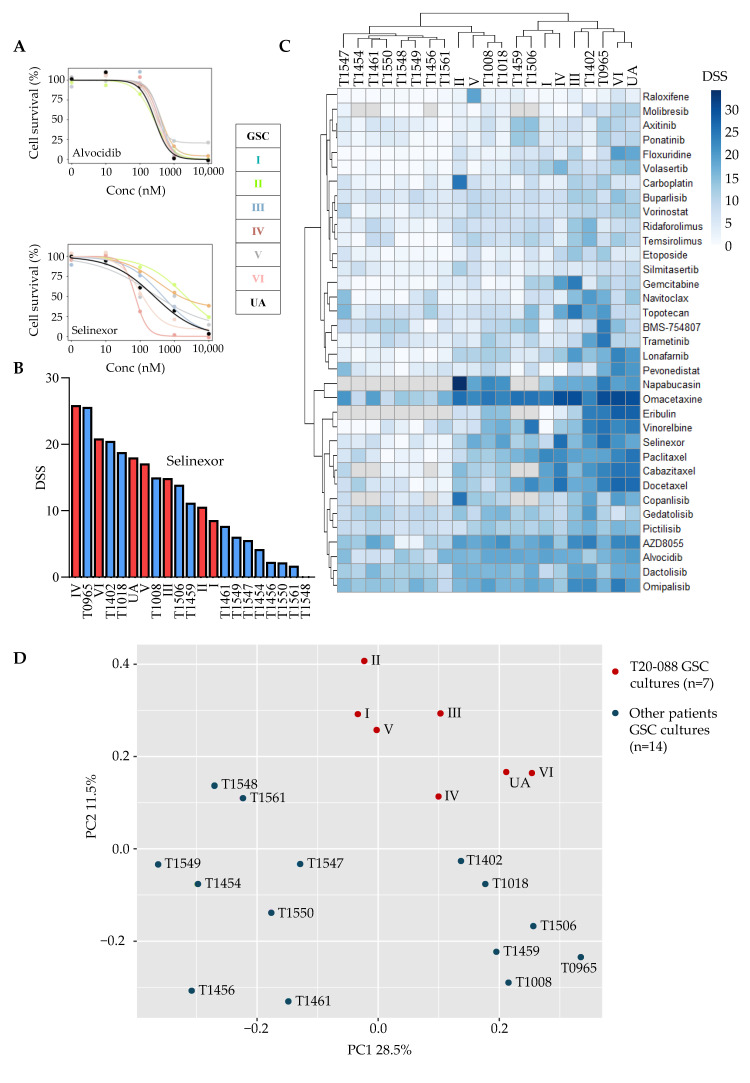
Multiregional GSC cultures (I–VI, UA) display heterogeneity in drug sensitivity but cluster separately from 14 other GSC cultures. (**A**) Dose–response curves demonstrating a homogeneous (alvocidib) and heterogeneous (selinexor) drug response. (**B**) The range of selinexor responses in the multiregional GSC cultures along with 14 other patients’ GSC cultures from IDH_wt_ GBMs. (**C**) Unsupervised hierarchical clustering of drug sensitivity in the multiregional-derived GSC cultures combined with GSC cultures from other patients. Drugs are selected by DSS ≥ 10 in at least one of the multiregional cultures. (**D**) Principal component analysis of GSCs. Although the difference is not very prominent, the proportion of drugs with high variation across samples is higher between the patient cultures compared to the multiregional cultures. In addition, the multiregional cultures are separated from the other samples only in the second principal component.

We next explored the drug sensitivity patterns in the multiregional GSCs to the 14 other GSC cultures (Supplementary Table S8). We found both homogenous and heterogeneous drug sensitivity patterns to individual drugs between the cultures. For example, the CDK inhibitor alvocidib had very similar and moderate sensitivity across all cultures, with a DSS ranging from 15.7 to 19.1 (Figure 4A). On the other hand, the sensitivity to the apoptotic modulator selinexor was more heterogeneous, with a DSS ranging from low (DSS 8.6) to high (DSS 25.9, Figure 4A). This heterogeneous sensitivity patterns of selinexor did, however, not exceed the DSS range between GSC cultures derived from 14 additional IDH^wt^ GBM patients (DSS range 0–25.6) (Figure 4B).

To compare the ITH to the intertumoral heterogeneity in drug sensitivity patterns between the group of cultures, we performed an unsupervised hierarchical clustering of the 35 drugs that displayed DSS ≥ 10 in at least one of the multiregional GSC cultures. The multiregional GSC cultures were found in both of two main clusters, reflecting cultures with either low or high drug sensitivity (Figure 4C). A similar pattern was also found in clustering analyses utilizing the entire drug panel (Supplementary Figure S2). Principal component analysis (PCA) of all of the GSC cultures (n = 21,) only including the drugs represented in all cultures (n = 103 drugs), showed that the multiregional GSC cultures clustered closer together than the other samples, indicating that overall intratumoral heterogeneity is smaller than intertumoral heterogeneity (Figure 4D). We further identified 34 drugs with significantly larger intertumor variance compared to intratumor variance (*p* < 0.05, FDR-adjusted) (Supplementary Figure S3).

Collectively, in terms of drug sensitivity, we observed that the multiregional GSC cultures clustered in two groups. These groups were mainly separated by a considerable difference in the number of drugs with a DSS ≥ 10 (medium to high drug responses). This heterogeneity, however, did not exceed the overall variation between individual tumors.

### 3.4. Tumor- and Culture-Specific Drug Sensitivity Patterns

**Figure 5:**
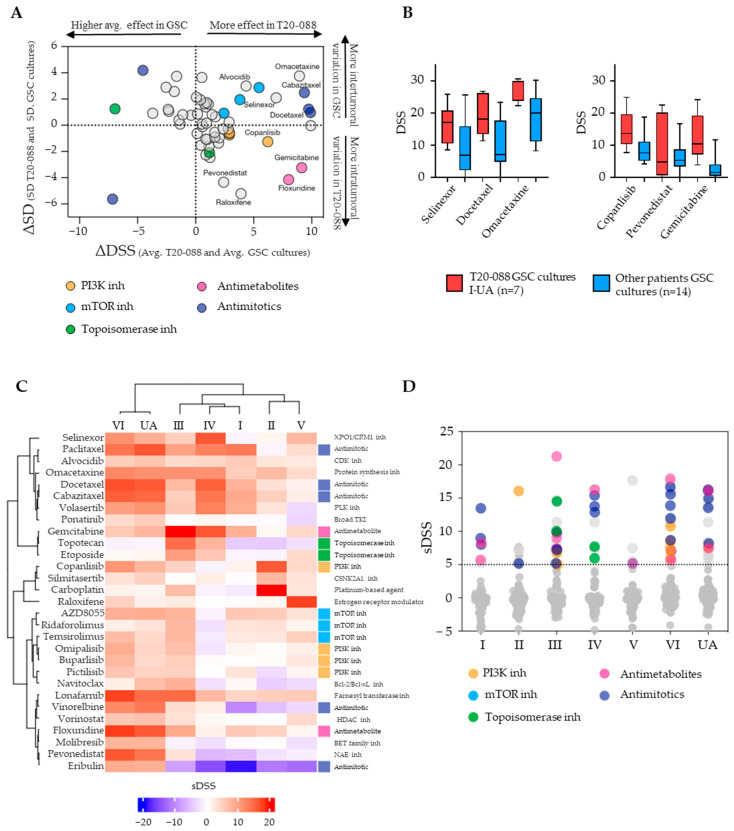
Identification of T20-088- specific drugs considering intra- and intertumoral heterogeneity. (**A**) Scatter plot displaying the drug sensitivity of single drugs in T20-088 compared to the average response in GSC cultures from 14 other patients with IDH_wt_ GBM. ΔDSS (X-axis) equals different effects in T20-088 from the average response in the other cultures. ΔSD (Y-axis) equals differences in the intra- versus intertumoral heterogeneity. (**B**) Examples of T20-088-selective drugs with low (left) and high (right) intratumoral variation in drug sensitivity patterns. (**C**) Unsupervised hierarchical clustering of the multiregional GSC cultures utilizing selective DSS (sDSS) to identify culture-specific drug sensitivity patterns. Drugs included in the heatmap are filtered by one of the multiregional cultures having a DSS ≥ 10 (n = 29 drugs). (**D**) sDSS of the multiregional GSC cultures at drug-class level demonstrating a more culture-specific drug sensitivity pattern.

To explore drugs specific for the tumor where we had performed multiregional sampling, we calculated the difference in average DSS in T20-088 to the average DSS of all GSC cultures. Further, we plotted ΔDSS against the differences in standard deviations of the two groups (ΔSD). This provided the identification of the drugs with increasing specificity towards the multiregional GSC cultures (positive ΔDSS, X-axis), along with the separation of drugs with either more homogeneous (positive ΔSD, e.g., omacetaxine or alvocidib) or heterogeneous (negative ΔSD, e.g., pevonedistat or copanlisib) responses compared to the other GSC cultures (Figure 5A). This allowed for the separation of drugs with a tumor-specific sensitivity to the multiregional GSC cultures into categories of broader activity across several multiregional GSC cultures (upper right quadrant) and more regionally culture-specific drug sensitivities (lower right quadrant). For example, the drugs selinexor, docetaxel, and omacetaxine were on average more effective in the multiregional GSC cultures, with less variation between the individual cultures compared to the other 14 GSC cultures from GBM (Figure 5B, left). In comparison, the drugs copanlisib, pevonedistat, and gemcitabine were similarly on average more effective in the multiregional GSC cultures than the other GSC cultures. However, these drugs displayed large culture-to-culture variation, indicating more intratumoral heterogeneous sensitivity patterns in selected drugs (Figure 5B, right). To identify culture-specific drug sensitivity patterns in individual multiregional GSC cultures, we calculated the differential drug response of the individual multiregional GSCs to the average response across the entire GSC cohort of treatment-naïve GBMs (selective DSS). This identified, for example, the relative increased sensitivity to topoisomerase inhibitors in culture III and IV compared to the rest, and the relative increased sensitivity to PI3K and mTOR inhibitors in culture III, VI, and UA compared to culture IV (Figure 5C). At the level of drug classes, we found, overall, various classes of drugs to be enriched in specific cultures from the multiregional sampling, such as conventional chemotherapies (antimitotics, antimetabolites, and topoisomerase inhibitors), kinase inhibitors (PI3K and mTOR inhibitors), and rapalogs (temsirolimus and ridaforolimus), demonstrating a more culture-specific drug sensitivity pattern (Figure 5D). This suggests that using a single and focal biopsy to establish patient-specific GSC cultures for drug sensitivity testing may underestimate the total complexity of drug sensitivity patterns within a tumor.

## 4. Discussion

In this study, we have described a molecular, cellular, and functional ITH in the GSC populations derived from multiregional biopsies. Our main findings were that these cultures in general shared phenotypic patterns, stem cell characteristics, and gene expression profiles. However, there were differences in cancer-relevant mutation profiles between the cultures and in sensitivity to anticancer drugs. These intratumoral differences, however, were less pronounced than between GSC cultures derived from other patients.

To our knowledge, this is the first study to describe molecular and functional ITH in GSC cultures derived from biopsies harvested at cm scale across the entire tumor mass. A few studies have explored ITH within GSC cultures derived from single cells [11,12,23]. In contrast to this study, their cell cultures were established from randomly sampled single cells dissociated from tumor tissue directly or from the parental multiclonal GCS culture, therefore unlikely to reflect the spatial range of the original tumor mass. In our study, we found that multiregional GSC cultures in general shared morphology, growth rate, and the ability to differentiate. Even in the GSC culture IV, which grew semi-adherently, we observed multiple spheres in the culture, and it differentiated like the other multiregional cultures. Likewise, Reinartz et al. [23] described a similar pattern comparing one parental multiclonal culture to GCS cultures derived from five single cells. They observed that all cultures were able to self-renew as spheres and differentiate into more mature phenotypes, despite some morphological differences between the clones. In contrast, Meyer et al. [11] found a wide and independent variation in proliferation and differentiation abilities in single cell GSC clones derived from the same GBM tumor. Also, Segerman et al. [12] showed that single cell GSC clones derived from the same GBM tumor varied in morphology and in terms of initial expansion rate and lag phase. A possible explanation for the ITH differences observed in multiclonal- versus single cell-derived GSCs may be that the underlying clonal heterogeneity is masked in the averaged output from multiclonal cultures.

We found numerous differences in exome aberrations between our cultures, including changes known to have significant impact in GBM. For example, we found that three of the seven multiregional GSCs harbored PTEN SNVs, while only one showed EGFR amplification. In Meyer et.al, they identified balanced/wild-type EGFR expression within one clone and increased expression within another clone and its multiclonal parental culture, while the rest of the clones displayed EGFR amplification—in total six GSC cultures derived from within the same tumor. Heterogeneity was also observed within 13 clones from another tumor in which one had EGFR amplification and the rest were balanced/wild type. Similarly, Reinartz et al. [23] found overall differences in global SNVs between clonal cultures, indicating ITH. Heterogeneity in single-cell mutations may become averaged when comparing multiclonal cultures. However, our seven multiregional GSC cultures were highly heterogeneous, indicating that significant heterogeneity exists in GSC mutation patterns across the tumor mass. More directly comparable to our study, Segerman et al. [12] compared two multiclonal cultures derived from the same tumor. In contrast to our findings, these two displayed identical mutational patterns including PTEN and EGFR aberrations. However, the spatial distribution of the original biopsies was not considered, and their identical genetic profile may have resulted from biopsies closely located.

The global gene expression in multiregional GSC cultures was generally highly correlated, with some significant differences in gene expression related to neuroactive ligands and receptors. The cultures derived from the focally harvested biopsies were more enriched in genes related to the mesenchymal than the proneural subtype. In contrast, UA displayed a lower expression of genes within the mesenchymal profile and a slightly increased enrichment in proneural genes. However, the heterogeneity between multiregional GSC cultures was strikingly lower than the heterogeneity between GSC cultures derived from other IDH^wt^ GBMs. Likewise, Meyer et al. [11] observed that transcription in clones from the same tumor were more similar to each other than between different tumors. When they calculated subtype scores (classical, mesenchymal, and proneural) for the clones from four tumors and their parental cultures, they found that the clones displayed high scores of more than one subtype for each tumor. A similar pattern was also demonstrated in Segerman et al. [12], showing a high degree of variability in the transcriptomes of clones derived from each tumor, although with less variation than between tumors. They also observed both the mesenchymal and proneural gene expression subtypes in clones within the same tumor. Further, and in line with our study, they found the transcriptomes of clones from two biopsies harvested from the same tumor to be highly similar. The heterogeneous gene expression pattern existing in single cells is likely masked in multiregional samples, only displaying the averaged gene expression levels.

When comparing drug sensitivity patterns of the multiregional cultures, three out of seven multiregional cultures displayed a significantly higher number of drugs with median to high responses compared to the other four cultures. An observed intratumoral variation in drug sensitivity is in accordance with previous reports on drug sensitivity in clonal GCS cultures [11,12]. Meyer et al. [11] screened drug efficacy in six to eight individual clones from each of three tumors to 98 drugs and found that every tested tumor showed clone by clone variability in responses to several drugs. Although exploring a smaller drug panel including only 15 drugs, Segerman et al. [12] also found extensive clonal variability within tumors, in which clones from one tumor exhibited heterogeneous responses comparable with the range seen between different parental cultures from different patients. Within all clonal libraries, a substantial proportion of the clones displayed more drug resistance compared to each of the clones’ corresponding parental culture, which displayed low to intermediate resistance. Clones resistant to one drug also tended to be resistant to most of the drugs in the panel, regardless of their mechanism of action. Thus, the ITH in drug sensitivities seems overall to be similar in clonal and multiregional GSC cultures.

The overall pattern of the multiregional GSC cultures across molecular, cellular, and functional analysis demonstrated some but not full consistency. The somatic mutations clustering VI and UA as a subgroup were only slightly reflected in global gene expression, as UA showed somewhat less correlation to the other cultures. In drug responses, culture VI and UA, but also III, were strikingly more drug sensitive. However, the drug response profile of III was different from VI and UA. The UA was harvested in a very different manner than focal biopsies and was likely to display some differences. In contrast to focal biopsies, the tissue was sampled by an ultrasonic aspirator used throughout the tumor resection and could potentially be more enriched in normal cells and tumor cells from the tumor periphery. A recent study focusing only on gene expression found UA-harvested tissue to be enriched in genes related to the leading edge and infiltrating zone in gliomas compared to focal biopsies [37]. However, we found UA and VI highly correlated in their drug sensitivity pattern, supporting the use of UA as a relevant source for GSC culture establishment.

The use of genomics to guide cancer therapies has been less successful than anticipated [38,39], and primary cell cultures such as GSC cultures are increasingly used for the identification of personalized, functional drug targets in clinical trials [24,25,40,41]. Often, small single-tumor biopsies are used as a source for the establishment of GSC cultures. Our findings indicate that studies including cultures derived from single biopsies only may suffer from an underestimation of the total molecular complexity. Three of the seven cultures that we investigated for functional drug sensitivity were more sensitive to anticancer drugs than the rest. Although we found some drugs with sensitivity across all GSCs, statistically significant heterogeneity between cultures was also seen. This heterogeneity may have important implications for the choice of targeted therapy if used clinically in personalized treatment protocols. Using the patient sample presented in this study to illustrate such a scenario, it is likely that drug selection from the screening of culture II would be quite different from culture VI. Thus, our findings suggest the use of multiple biopsies to represent the entire tumor mass in the development of GSC drug-screening treatment strategies.

A shortcoming in the present study is the use of a single tumor to describe ITH. This limits our ability to draw more general conclusions on multiregional functional and molecular heterogeneity in GBM. We have also investigated the ITH only in the GSC population within the tumor. Selective culturing to enrich the GSC population reduces the cellular complexity from the parent tumor that may influence the drug sensitivity patterns. Exploration of ITH in a more complex organoid model system is an alternative, but current models are still not suitable for high-throughput drug screening strategies [42,43,44].

## 5. Conclusions

Our study demonstrates that although the GSC cultures derived by multiregional sampling from the same tumor share phenotypic and tumor stem cell traits, they differ in terms of molecular makeup, mutational burden, and sensitivity to various anticancer drugs. This indicates a variation within the GSC pool of individual tumors. Our findings add functional intratumoral complexity to GBM biology and suggest the use of multiple biopsies to represent the entire tumor mass in the development of personalized treatment options targeting GSCs.

## Data Availability

Data are contained within article.

## Supplementary Materials

The following supporting information can be downloaded at: https://www.mdpi.com/article/10.3390/cancers15245826/s1. Figure S1: Spearman correlation in gene expression (FPKM) of multiregional GSC cultures and GSC cultures derived from 13 other patients’ IDH_wt_ GBM. Figure S2. Unsupervised hierarchical clustering of drug sensitivity patterns from all drugs (115) in multiregional sampled GSC cultures (left) compared to drug sensitivity pattern in GSC cultures from 14 other patients’ IDH_wt_ GBM (right). Figure S3. Proportion of drugs with greater intertumor heterogeneity compared to intratumor heterogeneity from *p*-value histogram. Table S1: List of drugs. Table S2: Flow cytometry data. Table S3: Mutations. Table S4: Heterogenous and homogenous genes. Table S5: KEGG pathways. Table S6: Temozolomide analysis. Table S7: Multiregional GSCs DSS IC50 and AUC. Table S8: All GSC’s Drug Sensitivity Scores (DSS).
