# Supplementary material for: Functional and Molecular Heterogeneity in Glioma Stem Cells Derived from Multiregional Sampling
Source: Cancers (Basel). 2023 Dec 13;15(24):5826. doi: 10.3390/cancers15245826 (PMC10741477; doi:10.3390/cancers15245826)

Supplementary Figure S1

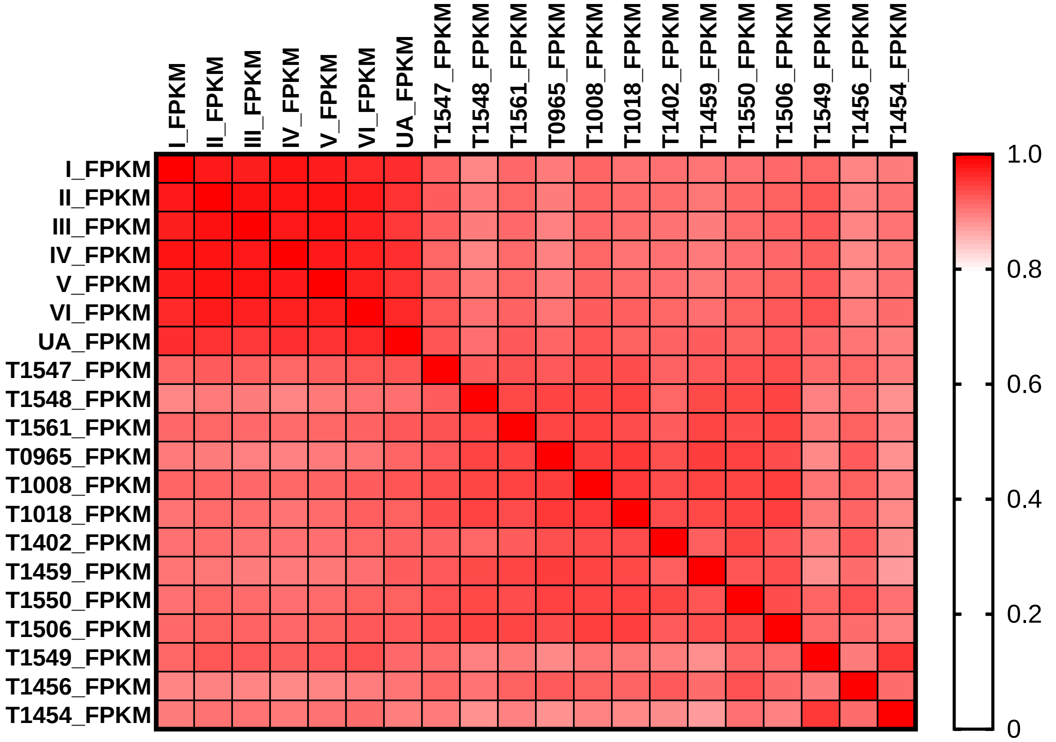

Spearman correlation Expression data

Supplementary Figure S2

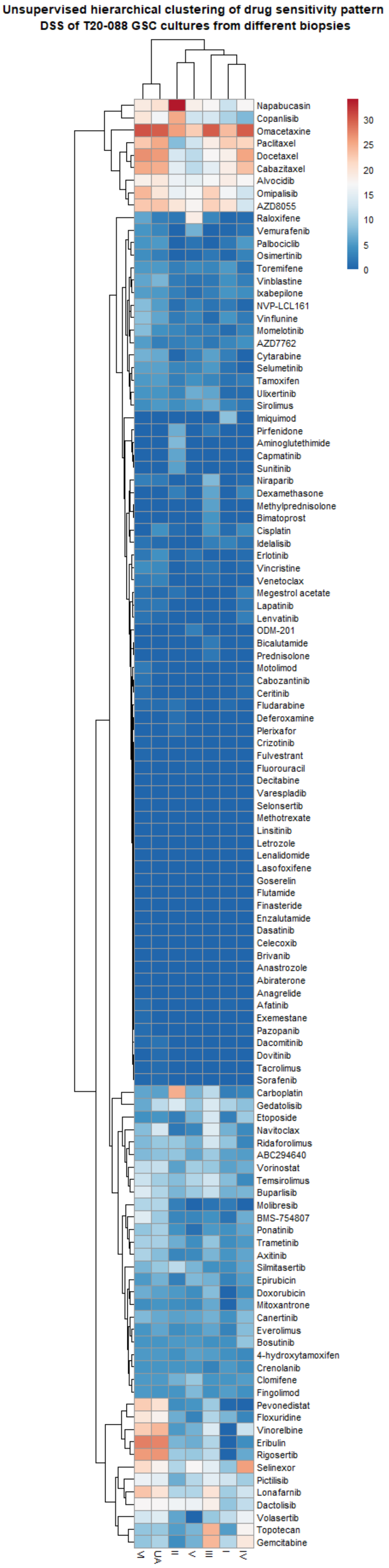

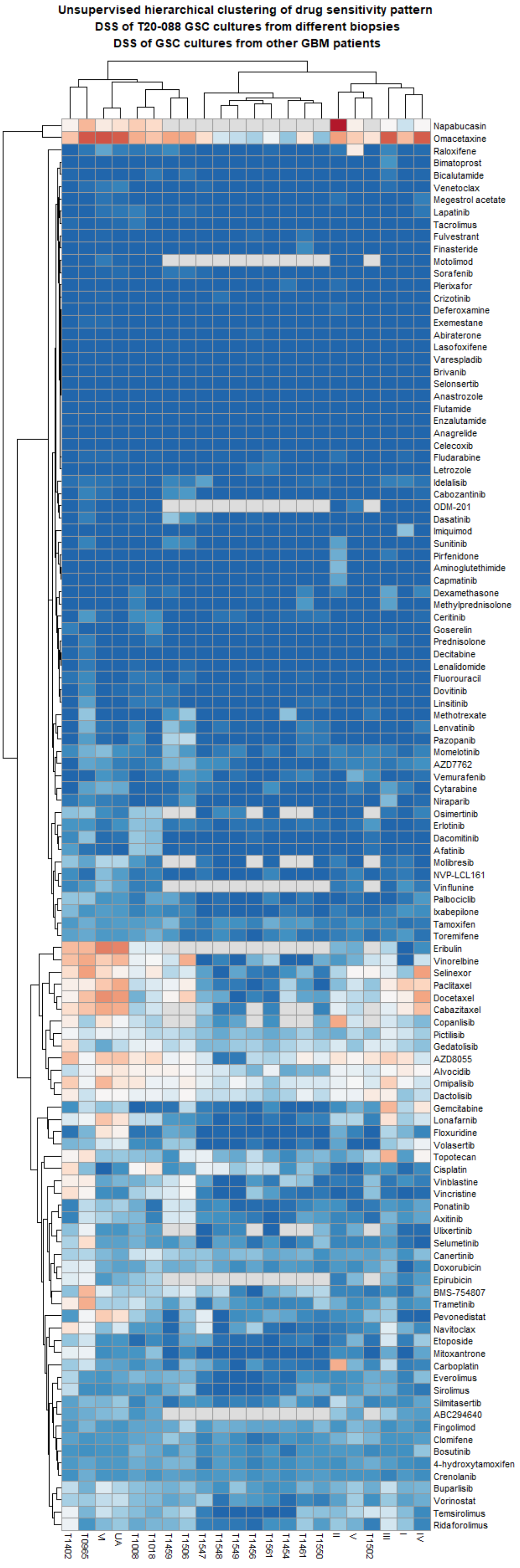

Supplementary Figure S3

ID	DRUG_NAME	F stastistic	p-value (raw)	pvalue (FDR-adjusted)
FIMM003716	Abiraterone	Inf	0	0
FIMM100387	Brivanib	Inf	0	0
FIMM003775	Dasatinib	Inf	0	0
FIMM000428	Finasteride	Inf	0	0
FIMM000257	Goserelin	Inf	0	0
FIMM100377	Lasofoxifene	Inf	0	0
FIMM003782	Lenalidomide	Inf	0	0
FIMM000290	Letrozole	Inf	0	0
FIMM003772	Linsitinib	Inf	0	0
FIMM000649	Methotrexate	Inf	0	0
FIMM003741	AZD7762	72.6	0.000017	0.00015
FIMM003786	Temsirolimus	61.8	0.000027	0.00022
FIMM003755	Everolimus	47.6	0.000059	0.00044
FIMM023795	Sirolimus	44.9	0.00007	0.00048
FIMM000491	Paclitaxel	38	0.000114	0.00074
FIMM003797	Docetaxel	33.6	0.000163	0.00099
FIMM023821	Ixabepilone	27.3	0.000296	0.00169
FIMM133902	Selinexor	26.6	0.000318	0.00171
FIMM003725	Alvocidib	21	0.000627	0.0032
FIMM133786	Lonafarnib	17.5	0.001061	0.00515
FIMM003778	Axitinib	16.9	0.001167	0.00539
FIMM003743	AZD8055	15.9	0.001387	0.00612
FIMM100376	Toremifene	15.2	0.001574	0.00664
FIMM023797	Floxuridine	13.7	0.002096	0.00847
FIMM023798	Gemcitabine	13.1	0.002345	0.00875
FIMM000370	Vinblastine	13.1	0.002346	0.00875
FIMM100379	Omacetaxine	11.5	0.003409	0.01205
FIMM023814	Etoposide	11.4	0.003479	0.01205
FIMM003707	Navitoclax	10.8	0.004042	0.01352
FIMM133892	NVP-LCL161	8.9	0.006673	0.02157
FIMM109441	Momelotinib	6.7	0.014141	0.04425

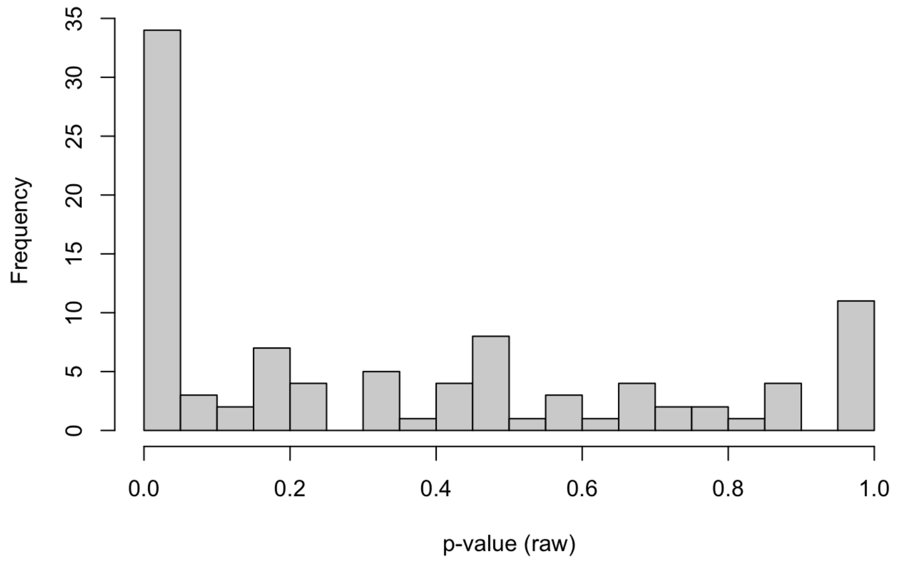

Supplement: Supplementary file 1 [file cancers-15-05826-s001.zip › cancers-2734281-supplementary/Cancers-2734281_Supplementary figures.pdf]
